# Effects of photobiomodulation therapy on inflammatory mediators in patients with chronic non-specific low back pain: Protocol for a randomized placebo-controlled trial

**DOI:** 10.1097/MD.0000000000015177

**Published:** 2019-04-12

**Authors:** Shaiane Silva Tomazoni, Leonardo Oliveira Pena Costa, Jon Joensen, Martin Bjørn Stausholm, Ingvill Fjell Naterstad, Ernesto Cesar Pinto Leal-Junior, Jan Magnus Bjordal

**Affiliations:** a Masters and Doctoral Programs in Physical Therapy, Universidade Cidade de São Paulo, São Paulo, Brazil; b Physiotherapy Research Group, Department of Global Public Health and Primary Care, University of Bergen, Bergen, Norway; c Laboratory of Phototherapy and Innovative Technologies in Health, Nove de Julho University; d Post-graduate Program in Rehabilitation Sciences, Nove de Julho University, São Paulo, Brazil.

**Keywords:** inflammatory mediators, low-level laser therapy, non-specific low back pain, photobiomodulation therapy

## Abstract

**Introduction:**

Low back pain (LBP) is ranked as one of the most prevalent health conditions. It is likely that some inflammatory mediators could be associated with pain and disability in these patients. Photobiomodulation therapy (PBMT) is a non-pharmacological therapy often used in patients with LBP and one of the possible mechanisms of action of therapy is modulate inflammatory mediators. However, to date there are no studies that evaluated the effects of PBMT on the levels of inflammatory mediators in patients with LBP. The aim of this study is to evaluate the acute effects of PBMT on systemic levels of inflammatory mediators and pain intensity in patients with chronic non-specific low back pain.

**Methods and analysis:**

This is a prospectively registered, two-arm randomized placebo-controlled trial with blinded patients, assessors and therapists. Eighteen patients with chronic non-specific LBP will be randomized into 2 groups: placebo or active PBMT. The treatment will be provided in a single session. The primary outcome will be levels of prostaglandin E_2_ (PGE_2_). The secondary outcomes will be levels of necrosis factor alpha (TNF-α), interleukin 6 (IL-6) and pain intensity. Biochemical and clinical outcomes will be measured at baseline and 15 minutes after the single treatment session.

**Discussion:**

Despite PBMT be used in musculoskeletal disorders such as LBP, to the best of our knowledge this is the first study that will investigate a possible biological mechanism behind the positive clinical effects of PBMT on non-specific chronic low back pain.

**Ethics and dissemination:**

The study was approved by the Regional Research Ethics Committee. The results will be disseminated through publication in peer-reviewed international journal and conferences.

**Trial registration number:**

NCT03859505.

## 1 Introduction

Non-specific low back pain is a complex and extremely frequent condition^[1]^ strongly associated with high levels of disability.^[2]^ Some aspects can be associated with low back pain, and the identification of these aspects is helpful in both prevention and management of this condition.^[3]^ One of these aspects is the possible association between inflammatory mediators and low back pain, since the increased of pro inflammatory cytokines levels is often observed in painful conditions.^[4,5]^

Evidences have demonstrated increase of pro inflammatory cytokines levels in patients with low back pain. Wang et al^[6]^ demonstrated increase of plasmatic levels of necrosis factor alpha (TNF-α) in patients with chronic low back pain when compared to healthy individuals, for example. However, no significant correlation of TNF-α levels to pain intensity or disability were identified. Moreover, it was observed that there is an increase of TNF-α ^[7,8]^ and interleukin (IL) 6 levels in patients with acute low back pain.^[8]^ Thus, the presence of some inflammatory mediators might be associated with pain and disability in patients with low back pain, since pro inflammatory cytokines such as IL-6, TNF-α and IL-1 contribute to the activation of nociceptors that generate potential of action and pain hyper sensibility.^[4,5]^

Several nonpharmacologic and noninvasive management options are available for patients with low back pain (e.g., exercises, electrophysical agents and manual therapy).^[9]^ However, the mechanisms of action of available therapies to reduce pain and disability in these patients still remain uncertain. According to the abovementioned studies,^[6–8]^ the modulation of inflammatory mediators levels could be considered a possible strategy in the low back pain management and thus, the use of photobiomodulation therapy (PBMT) could be interesting in this case.

PBMT is a non-pharmacological and non-thermal intervention that consists in applying a non-ionized form of light, which includes LASER (light amplification by stimulated emission of radiation) and LED (light-emitting diodes) from visible to infrared spectrum.^[10]^ PBMT is often used in the treatment of musculoskeletal disorders such as low back pain;^[11,12]^ however, the exact mechanism of action through PBMT decreases pain intensity and disability in these patients is still unknown. Evidences suggest that PBMT is effective in modulate inflammatory mediators such as TNF-α, IL-1β, IL-6, IL-10 and prostaglandin E_2_ (PGE_2_), both in experimental and clinical studies,^[13–18]^ contributing to pain relief^[18,19]^ and tissue repair.^[20,21]^ The biological responses of PBMT on these inflammatory mediators may be targeted as possible mechanism of action for its beneficial clinical effects.

However, currently there are no studies about the effects of PBMT on inflammatory mediators in patients with non-specific low back pain, investigating whether this could be a possible mechanism of action. Therefore, the aim of this study is to evaluate the acute effects of PBMT on systemic levels of inflammatory mediators (TNF-α, IL-6 and PGE_2_) and pain intensity in patients with chronic non-specific low back pain.

## 2 Methods and analysis

### 2.1 Design

A two-arm, parallel randomized, triple-blinded (patients, therapists and outcome assessors), placebo-controlled trial will be performed. The protocol of this study has been prospectively registered on Clinicaltrials.gov (NCT03859505). The study will be conducted at Department of Global Public Health and Primary Care, University of Bergen, Norway.

### 2.2 Ethical aspects

The present study will follow the ethical guidelines and was approved by the Regional Research Ethics Committee (Protocol No. 2018/1361/REK nord). Patients will be informed about all study procedures and asked to sign the Informed Consent Form prior to their enrollment in the study. Research personnel will take all appropriate and customary steps to ensure that data remain secure and that patient privacy and confidentiality will be maintained.

### 2.3 Characterization of sample

Since no studies assessing the effects of PBMT on inflammatory mediators in patients with non-specific low back pain are available, the number of patients per group in the present study was calculated based on a pilot study. This pilot study was recently conducted by our research group with 3 patients per group in order to estimate the sample size. A β value of 20% and an α of 5% were used to calculate the sample size. The pilot study showed that applying PBMT in patients with non-specific low back pain resulted in levels of PGE_2_ (primary outcome of the present study) of 1.05 pg/μl (0.42 standard deviation), whereas applying the placebo in patients with non-specific low back pain resulted in levels of PGE_2_ of 1.52 pg/μl (0.39 standard deviation). We used the Researcher's Toolkit to calculate the sample size (https://www.dssresearch.com/resources/calculators/sample-size-calculator-average). Based on the aforementioned parameters used to calculate the sample, we found a sample of 9 patients per group, for a total of 18 patients. Since the phototherapy device used in the study causes no harmful thermal effects,^[22]^ patients of different skin colors will be recruited. The patients will be recruited through primary care doctors and physiotherapists.

### 2.4 Eligibility criteria

Inclusion criteria:

- Patients with chronic non-specific low back pain (pain or discomfort between the costal margins and inferior gluteal folds with or without referred pain to the lower limbs);
- Persistent low back pain for at least 3 months;^[23]^
- Pain intensity of at least 3 points, measured by Pain Numerical Rating Scale;^[24]^
- Aged between 18 and 65 years;
- Both genders.

Exclusion criteria:

- Patients with severe skin diseases (e.g., skin cancer, erysipelas, severe eczema, severe dermatitis, severe psoriasis and severe hives lupus);
- Patients with low back pain associated with nerve root compromise (measured by clinical examination of dermatomes, myotomes and reflexes);^[25,26]^
- Serious spinal pathologies such as fractures, tumors, inflammatory and infectious diseases;
- Serious cardiovascular or metabolic disorders;
- Previous spinal surgery;
- Pregnancy.

### 2.5 Procedures

**Figure 1:**
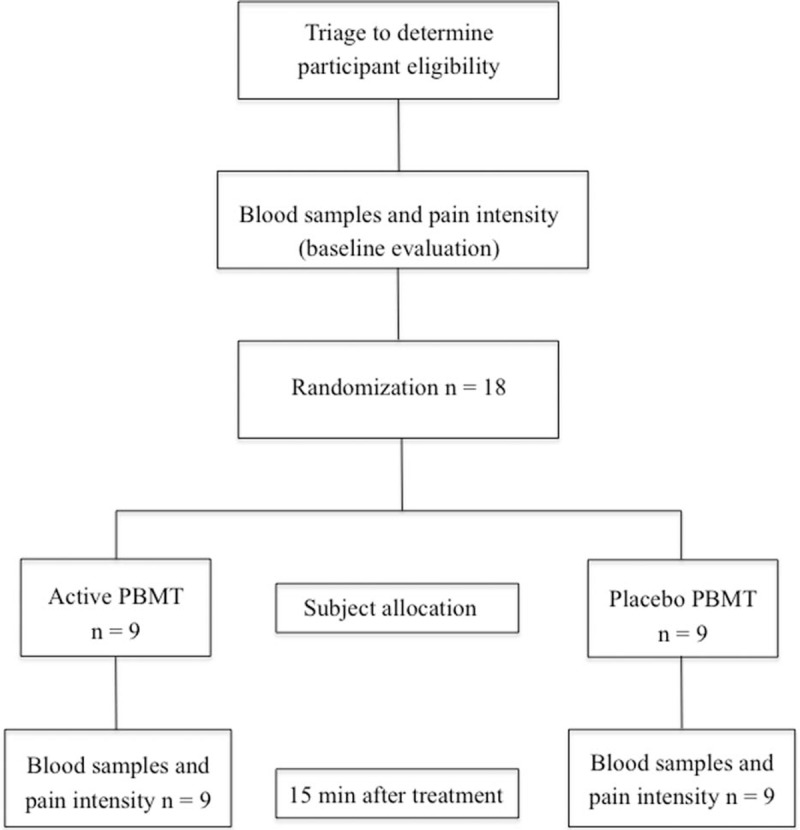
CONSORT flowchart.

The patients will be welcomed by the study's blinded assessor who will determine whether they will be eligible to participate in the study. Subsequently, a file will be completed with the patient's sociodemographic data and clinical history. Next, the primary and secondary outcomes of the study will be collected and all eligible patients will be randomized and allocated into 2 interventions groups: active PBMT or placebo PBMT. Fifteen minutes after the end of the single treatment session, the primary and secondary outcomes of the study will be collected again. The Consolidated Standards of Reporting Trials flowchart summarizing experimental procedures and patients is shown in Figure 1.

### 2.6 Randomization and blinding procedures

Prior to the single treatment session, patients will be randomized into their respective intervention groups: placebo or active PBMT. The randomization will be generated by a computer program (Excel Office 2010) and performed by a participating researcher not involved with the recruitment or evaluation of patients. This same researcher will be responsible for programming the PBMT device according to the result of the randomization (active or placebo mode) and will be instructed not to disclose the programmed intervention to the therapist, any of the patients or other researchers involved in the study until its completion. Concealed allocation will be achieved through the use of sequentially numbered, sealed and opaque envelopes. To ensure blinding for therapists and patients, the device will emit the same sounds and the same information on the display regardless of the programmed mode. Thus, patient and therapist will be blinded throughout the treatment.

### 2.7 Interventions

Patients will undergo the treatment (active PBMT or placebo), according to prior randomization, in a single session:

1. Active PBMT: The PBMT will be performed using the Multi Radiance Medical Super Pulsed Laser MR4 console (Solon, OH), with the SE25 (emitter with an area of 4 cm^2^) and LaserShower (emitter with an area of 20 cm^2^) cluster probes as emitters. Nine sites will be irradiated on the patient's lumbar region: 3 central sites on top of the spinous processes (between T11 and T12, L2 and L3, L5 and S1), using the SE25; in the same direction, but laterally, 3 sites on the left and 3 on the right (on the paravertebral muscles), using the LaserShower (LS). PBMT irradiation sites and parameters were chosen based on a previous study.^[27]^ The treatment will be performed in a single session and the patients will receive a total dose of 220.05 J. Table 1 shows PBMT parameters.
2. Placebo PBMT: Placebo PBMT will be delivered using the same device and the irradiated sites will be the same that active PBMT, but without any emission of therapeutic dose. Patients will receive a total dose of 0 J in placebo mode. The treatment will be performed in a single session.

**Table 1:**
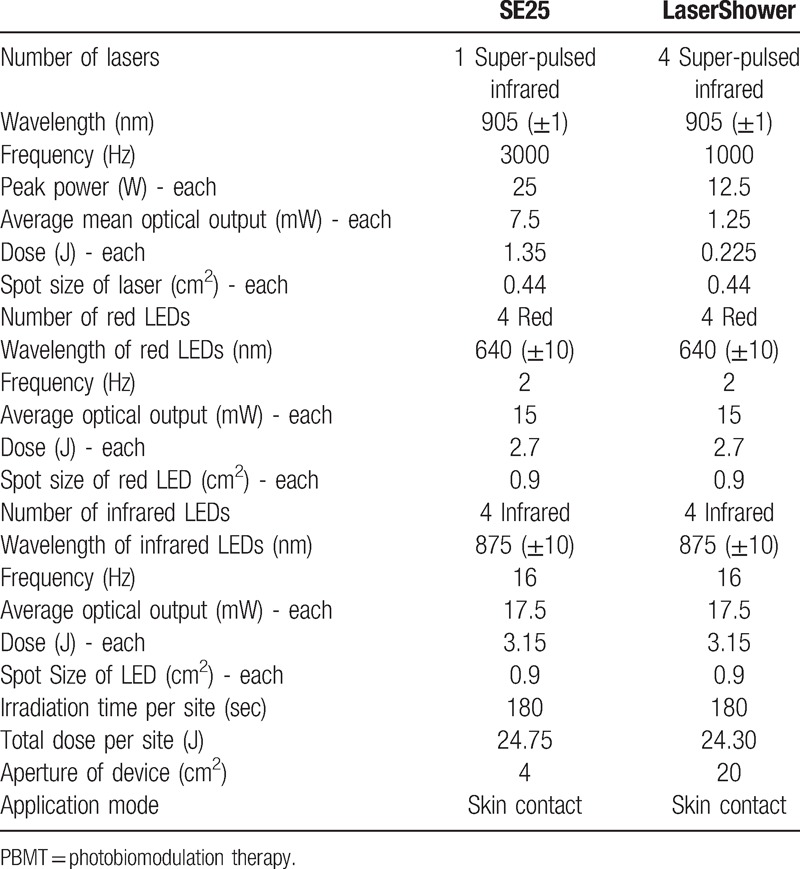
PBMT parameters.

### 2.8 Outcomes

The primary outcome (levels of PGE_2_) and secondary outcomes (levels of TNF-α and IL-6 and pain intensity) of the study will be obtained at baseline and 15 minutes after the single treatment session by an assessor who will not be aware of patients’ allocation to their treatment groups:

- Levels of TNF-α, IL-6 and PGE_2_ (inflammatory mediators): measured by enzyme-linked immunosorbent assay (ELISA) method. Blood samples (10 ml) will be collected by a qualified nurse (blinded to group allocation) and will be obtained from the antecubital vein. One hour after collection, each sample will be centrifuged at 3000 rpm for 20 minutes. Pipettes will be used to transfer the serum to Eppendorf tubes, which will be stored at −80°C until analysis. The levels of TNF-α, IL-6 and PGE_2_ in the blood samples will be determined by ELISA, using a commercial kit and following the manufacturer's instructions (BD Biosciences, USA). Spectrophotometric readings will be performed in a SpectraMax Plus 384 Absorbance Plate Reader (Sunnyvale, CA) with 450-nm wavelength and correction to 570 nm. The results of TNF-α and IL-6 will be expressed in pg/ml and the results of PGE_2_ will be expressed in pg/μl.
- Pain intensity: measured by the Pain Numerical Rating Scale.^[24]^ Pain Numerical Rating Scale evaluates pain intensity levels perceived by the patient on an 11-point scale ranging from 0 to 10, with 0 being ‘no pain’ and 10 ‘the worst possible pain.^[24]^ Patients will be instructed to score the level of pain intensity based at the time of evaluation.

### 2.9 Statistical analysis

The statistical analysis will be conducted following the principles of intention-to-treat analysis.^[28]^ The findings will be tested for their normality using the Shapiro-Wilk test. Parametric data will be expressed as mean and standard deviation and non-parametric data as median and respective upper and lower limits. Parametric data will be analyzed by two-way repeated measures analysis of variance (ANOVA, time vs experimental group) with post-hoc Bonferroni correction. Non-parametric data will be analyzed using the Friedman test and, secondarily, the Wilcoxon signed-rank test.

### 2.10 Data availability

The datasets generated and analyzed during the current study will be available from the corresponding author on reasonable request.

## 3 Discussion

Despite PBMT be used in musculoskeletal disorders such as low back pain, to the best of our knowledge this is the first study that will investigate a possible biological mechanism behind the positive clinical effects of PBMT on non-specific chronic low back pain. We strongly believe that this investigation can be helpful in the management of this condition.

Non-specific low back pain is a complex health condition and several treatment options may be used for symptoms relief.^[29]^ Over the years, it has been believed that some subgroups of this population can benefit from specific treatments, responding favorably to specific therapies.^[30,31]^ In this way, knowing the mechanism of action of available therapies becomes an important aspect in this process.

PBMT triggers positive effects acting in different ways, including increase of microcirculation,^[32]^ cytochrome c oxidase modulation and ATP synthesis,^[33]^ besides modulating some inflammatory biomarkers.^[17,18]^ Thus, if PBMT is able to modulate inflammatory mediators in non-specific low back pain, patients with increased levels of these mediators might be more responsive and consequently benefit more with the use of therapy.

It is important to highlight that the present study is innovative and present high methodological quality (i.e., is a randomized controlled trial, triple blinded and prospectively registered). The sample size was calculated to provide the appropriate statistical power to detect precise differences for the primary outcomes of the study; however, the sample size has not powered enough to be correlated with the secondary outcome (pain intensity). One of the limitations of the study is that although the PBMT parameters were based on previous studies, there was no optimization and only one dose will be tested.

### 3.1 Dissemination policy

The study will be disseminated through publication in peer-reviewed international journal and conferences.

## Author contributions

SST, LOPC, ECPLJ and JMB contributed to the concept and design of the study, established the hypothesis and wrote the original proposal. SST, LOPC, JJ, MBS, IFN, ECPLJ and JMB contributed significantly in creating the manuscript. ECPLJ and JMB performed critical revisions of the manuscript. SST wrote the final version of the manuscript. All authors read and approved the final version of the manuscript.

**Conceptualization:** Shaiane Silva Tomazoni, Leonardo Oliveira Pena Costa, Jon Joensen, Martin Bjørn Stausholm, Ingvill Fjell Naterstad, Ernesto Cesar Pinto Leal-Junior, Jan Magnus Bjordal.

**Funding acquisition:** Shaiane Silva Tomazoni.

**Investigation:** Ernesto Cesar Pinto Leal-Junior.

**Methodology:** Shaiane Silva Tomazoni, Jon Joensen, Martin Bjørn Stausholm, Ingvill Fjell Naterstad, Ernesto Cesar Pinto Leal-Junior.

**Project administration:** Shaiane Silva Tomazoni, Jon Joensen.

**Supervision:** Leonardo Oliveira Pena Costa, Jan Magnus Bjordal.

**Writing – original draft:** Shaiane Silva Tomazoni, Leonardo Oliveira Pena Costa, Ernesto Cesar Pinto Leal-Junior, Jan Magnus Bjordal.

**Writing – review & editing:** Shaiane Silva Tomazoni, Leonardo Oliveira Pena Costa, Ernesto Cesar Pinto Leal-Junior, Jan Magnus Bjordal.
